# Focused ultrasound ventro-oral thalamotomy alters cortical excitability and motor control in focal hand dystonia

**DOI:** 10.1093/braincomms/fcag403

**Published:** 2026-10-23

**Authors:** Joel Maamary, James Peters, Isabelle Osborne, Kain Kyle, Nicholas Olsen, Diane Ruge, Benjamin Jonker, Yael Barnett, Stephen Tisch

**Affiliations:** Department of Neurology, St Vincent’s Health Network, Darlinghurst, NSW 2010, Australia; School of Medical Sciences, The University of New South Wales, Randwick, NSW 2031, Australia; Department of Neurology, St Vincent’s Health Network, Darlinghurst, NSW 2010, Australia; School of Medical Sciences, The University of New South Wales, Randwick, NSW 2031, Australia; Department of Neurology, St Vincent’s Health Network, Darlinghurst, NSW 2010, Australia; The University of Sydney, Camperdown, NSW 2050, Australia; GE Healthcare Technologies Inc, Mascot, NSW 2020, Australia; Stats Central, Mark Wainwright Analytical Centre, University of New South Wales, Randwick, NSW 2031, Australia; Independent Research Scholar, Montpellier, France; School of Medical Sciences, The University of New South Wales, Randwick, NSW 2031, Australia; Department of Neurosurgery, St Vincent’s Health Network, Darlinghurst, NSW 2010, Australia; School of Medical Sciences, The University of New South Wales, Randwick, NSW 2031, Australia; Department of Radiology, St Vincent’s Health Network, Darlinghurst, NSW 2010, Australia; Department of Neurology, St Vincent’s Health Network, Darlinghurst, NSW 2010, Australia; School of Medical Sciences, The University of New South Wales, Randwick, NSW 2031, Australia

**Keywords:** high-intensity focused ultrasound, focal hand dystonia, transcranial magnetic stimulation, neuroplasticity, motor programme

## Abstract

Focal hand dystonia is a disabling disorder characterized by task-specific abnormal involuntary movements that disrupt motor control. While the aetiology is unknown, abnormalities in sensory processing, sensorimotor organization, and maladaptive plasticity are thought to contribute to pathogenesis, with both genetic and environmental factors implicated. Current first-line treatments may provide only partial or transient relief. High-intensity focused ultrasound offers a means of focally ablating the target pathways implicated in dystonia. The ventro-oral complex, a relay within the motor thalamus, represents a key node in the corticobasal gangliathalamocortical circuit involved in directing motor output. Surgical radiofrequency lesioning of the Vo-complex confers sustained benefit in focal hand dystonia however the mechanisms that underlie this improvement remain unexplored. Pallidal intervention, via deep brain stimulation, has been shown to modify abnormal cortical motor plasticity in individuals with generalized dystonia, and this modification has been correlated with symptom improvement. It has been hypothesized that, to obtain long-term symptom improvement in focal hand dystonia, similar permanent network or circuit-based modification is required. In this prospective single-arm interventional study, we analysed the clinical and neurophysiological correlates of 11 individuals who underwent high-intensity focused ultrasound ventro-oral complex thalamotomy during 12 months follow-up. These measurements allowed us to identify the immediate and delayed impacts of ventro-oral thalamotomy on symptom burden, motor performance, motor cortex excitability, spatial specificity, and associative plasticity in individuals with focal hand dystonia. These findings were compared to 10 age and sex-matched healthy control participants. Consistent with our hypothesis, ventro-oral complex thalamotomy was associated with symptomatic improvement in focal hand dystonia as measured by standardized rating scales. There was no long-term loss of strength or motor dexterity in the treated limb, although adverse events were common and persisted in two participants. Inducible abnormalities in motor cortex excitability were attenuated following treatment and the spatial specificity of motor cortical responses shifted towards control values. Stimulus response curve analysis demonstrated modification of neuroplastic gain, echoing effects previously reported for pallidal stimulation in generalized dystonia. We concluded that ventro-oral complex thalamotomy may be associated with symptom improvement in focal hand dystonia and is associated with identifiable changes in motor cortex excitability. This work presents an opportunity for further studies to build on the identified mechanistic insights into single target lesioning, and advance the field of personalized circuit-informed strategies for the treatment of movement disorders.

## Teaser

Maamary *et al.* report that MRI guided focused ultrasound ventro-oral complex thalamotomy was associated with symptom improvement in focal hand dystonia and was accompanied by changes in cortical excitability and associative plasticity. These findings provide possible mechanistic insights into the effects of targeted thalamic lesioning in dystonia.

## Graphical Abstract

**Graphical Abstract:**
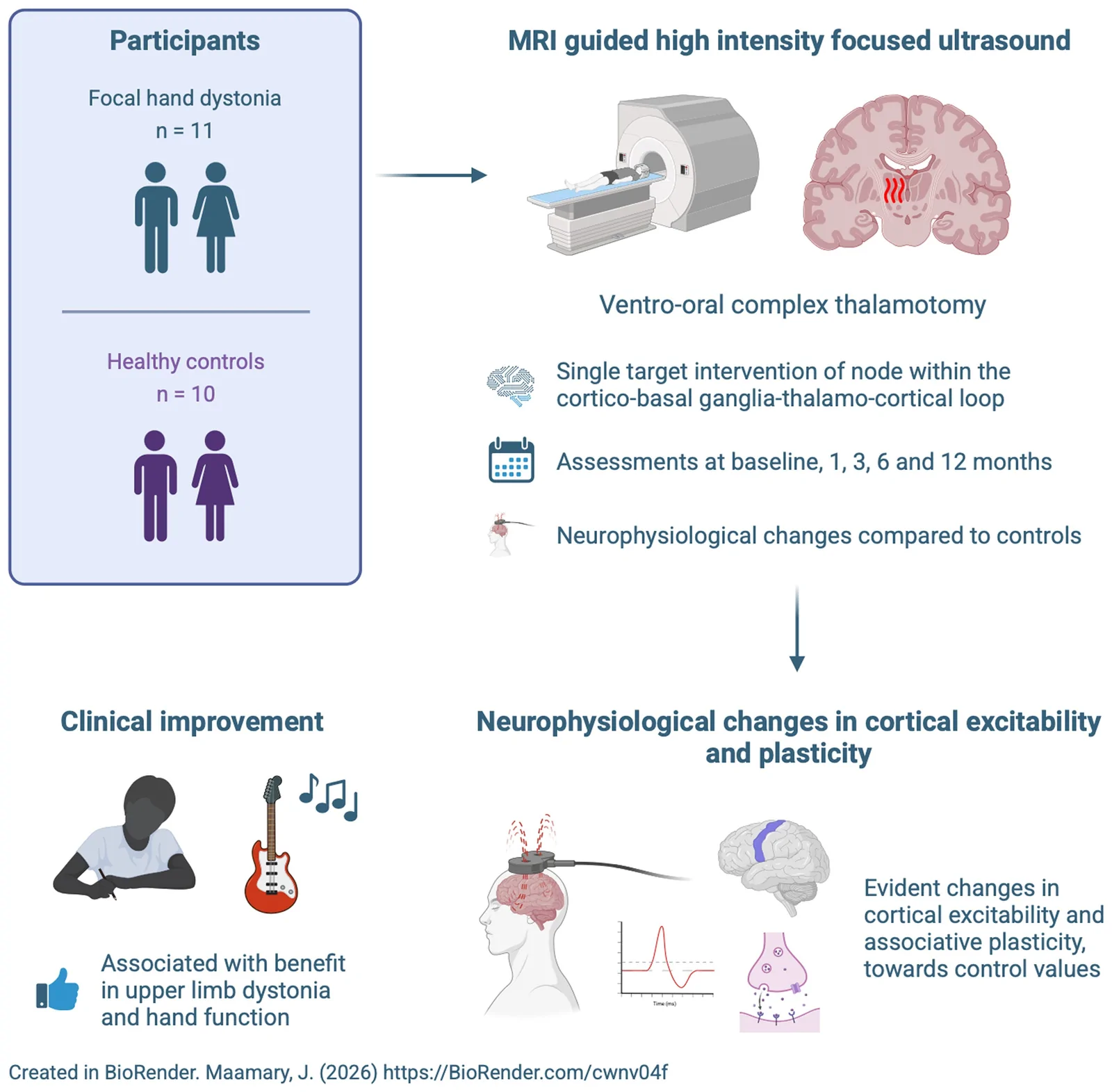

## Introduction

Focal hand dystonia is a movement disorder that is characterized by sustained or intermittent abnormal movements or postures that are isolated to the upper limb.^1^ In many cases, this is a ‘task-specific’ form of dystonia that is induced by performance of a particular motor pattern, thereby interfering with skilled task performance while sparing the affected body segment at other times. Despite evidencing generally preserved strength and gross motor function, individuals with focal hand dystonia experience profound impairments in fine motor control during highly learned behaviours, such as handwriting (writer’s cramp) or playing a musical instrument (musician’s dystonia). Although the pathophysiology of these conditions is incompletely understood, converging evidence implicates abnormalities in sensorimotor processing, excessive excitation, impaired inhibition and maladaptive plasticity as key contributors in disease expression. Additionally, abnormal cortical sensorimotor representations have been demonstrated in both animal models^2,3^ and in individuals with focal hand dystonia,^4^ suggesting disrupted synaptic organization within motor cortical networks. These disturbances may distort how the brain encodes, refines, and accesses movement, predisposing affected individuals to aberrant motor learning and task performance.

Excessive excitability and attenuated inhibitory responses are evident across multiple levels of the motor system in focal hand dystonia,^5,6^ encompassing not only the cortex but also the subcortical structures, the brainstem and the spinal cord.^7,8^ The execution of highly skilled learned movements requires a finely tuned balance between excitatory and inhibitory signalling, conceptualized within the ‘centre-surround’ model of motor control.^9-11^ Overexcitation, or loss of inhibitory signalling, may permit the emergence of unwanted muscle activity during task performance. Subsequently, in the presence of maladaptive neuroplastic responses, these unwanted movements become reinforced and consolidated into the motor programme (likened to a pathological motor *engram*),^12^ contributing to a task-specific engrained dystonic motor pattern.

Transcranial magnetic stimulation (TMS) studies have probed associative plasticity within the motor system in individuals with focal hand dystonia.^13-18^ Paired associative stimulation (PAS), using repeated activation of peripheral somatosensory afferents, is used to enhance synaptic efficacy through long-term potentiation (LTP)-like mechanisms, and this response is exaggerated in individuals with focal hand dystonia, compared to healthy controls.^14,16^ PAS induces time-dependent plastic responses through repeated coupling of peripheral nerve stimulation and cortical TMS, demonstrating heightened and poorly spatially regulated cortical responses in dystonia.^16,19^ Modification of abnormal cortical plasticity has been linked to clinical improvement following globus pallidus internus (GPi) deep brain stimulation (DBS) for generalized dystonia,^20^ suggesting that the modulation of maladaptive plasticity may play a role in symptom relief.

Causal evidence linking thalamic relay function to maladaptive plasticity in focal hand dystonia is limited. Lesioning of the thalamus, via ventral intermediate nucleus (VIM) thalamotomy, is an established and effective treatment of medically refractory tremor^21,22^ and demonstrates that targeted disruption of pathological circuit activity can yield sustained clinical benefit in movement disorders. In focal hand dystonia, both DBS and lesioning of the ventro-oral (Vo) complex are associated with symptom improvement.^23-28^ The Vo-complex acts as a crucial node in the cortico-basal gangliathalamocortical loop, implicating pallidothalamic relay pathways in disease expression. The mechanisms that underlie symptom improvement with Vo-complex intervention are not yet understood.

Magnetic resonance imaging (MRI)–guided focused ultrasound is a significant advancement in the field of stereotactic and functional neurosurgery, allowing precise, incisionless lesioning of deep brain structures with real-time imaging guidance. Lesioning of the Vo-complex, via high-intensity focused ultrasound (HiFU), allows for selective disruption of the pallidothalamic relay whilst minimizing the risk of off-target effects, presenting a unique opportunity to interrogate thalamic relay function in individuals with focal dystonia. A recent pilot study of HiFU Vo-complex thalamotomy^23^ demonstrated sustained improvements in the symptoms of writer’s cramp and musician’s dystonia at twelve months, establishing the feasibility of HiFU thalamic lesioning in focal hand dystonia. In this present study, we combined comprehensive longitudinal clinical assessments, using patient-reported outcomes and objective measures of strength, dexterity and coordination, with detailed neurophysiological measurements of cortical excitability and associative plasticity. This integrated approach was designed to determine not only whether HiFU Vo-complex thalamotomy led to an improvement in the symptoms of focal hand dystonia but also to quantify the presence of any motor cost associated with the procedure and identify whether clinical improvement is accompanied by measurable alterations in cortical excitability and plasticity. We hypothesized that focal disruption of the Vo-complex would result in symptomatic improvement and modification of PAS-induced cortical plasticity, thereby providing evidence for a causal role of thalamic relay signalling in the maintenance of the pathological motor programme. The association of these findings is important to support the concept that Vo-complex thalamotomy may allow for one to reset, or *forget*, a reinforced abnormal motor programme.

## Materials and methods

### Participants

A prospective clinical trial evaluating the safety and efficacy of HiFU Vo-complex thalamotomy in focal dystonia was approved by the St Vincent’s Health Australia Human Research Ethics Committee (HREC Ref: 2022/ETH00778) and registered with the Australia New Zealand Clinical Trials Registry (ACTRN12622000775718p). The study protocol has been published previously.^29^

A total of eleven individuals with a diagnosis of focal hand dystonia were enrolled between February 2023 and March 2024. One participant was withdrawn after the 3-month follow-up timepoint for reasons unrelated to the intervention or study procedures. An additional participant was subsequently recruited, resulting in a final cohort of eleven participants, ten of whom completed the planned 12-month follow-up period. All participants were assessed at baseline using standardized clinical scales and neurophysiological assessments. These evaluations were repeated at 1-, 3-, 6-, and 12-month follow-up, with the 6-month timepoint omitted for neurophysiological measures. The cohort included individuals with writer’s cramp, musician’s dystonia, or both. Ten healthy age- and sex-matched control participants were recruited for comparison of strength, dexterity and neurophysiologic measures.

### High-intensity focused ultrasound ventro-oral-complex thalamotomy

The details of the HiFU procedure at our institution have been previously published.^30^ On the day of treatment, participants underwent head shave and the administration of local anaesthetic scalp nerve blocks prior to application of a stereotactic head frame. An elastic coupling diaphragm was then placed over the scalp, affixed to the ultrasound transducer prior to being filled with cooled, degassed water. Participants were then positioned within a 3-Tesla MRI scanner (SIGNA Architect, GE Medical Systems, Milwaukee) and 3D T1 weighted imaging was performed for localization of the Vo anterior and Vo posterior nuclei in conjunction with preprocedural imaging (FGATIR). The Vo-complex was targeted in all participants using standard stereotactic coordinates,^31^ with an initial target 12 mm lateral to the midline, 2–2.5 mm superior to the anterior-posterior commissural (AC-PC) plane, and at the mid-commissural point (on average, approximately 12 mm AC to PC). Based on intra-procedural clinical response, the lesion was extended posteriorly to a final position 9 mm AC to the PC. 3.5–4 mm of clearance from the internal capsule was required to avoid damage to the corticospinal motor fibres.

Lesions were created using an Exablate Neuro system (Insightec, Tirat Carmel, Israel) equipped with a 650-kHz, 1024-element phased-array ultrasound transducer. Initial low-power sonications were performed to confirm accurate targeting in three orthogonal planes, producing mean focal temperatures of 40–45°C, monitored using MR thermography. Following confirmation of accurate focus, therapeutic sonications were delivered with stepwise increases in acoustic energy while continuously monitoring both maximal and average focal temperatures at the target. Between sonications, participants underwent neurological examination and were asked to perform their dystonia-inducing task to assess symptom provocation, on-table clinical benefit, and the emergence of adverse effects. All equipment used during task performance was MRI-compatible and non-ferromagnetic.

### Clinical assessments

Clinical outcomes were assessed using standardized rating scales appropriate to the dystonia subtype. These included the Arm Dystonia Disability Scale (ADDS; 0–100% ability scale, where 100% equates to no disability),^32^ the Writer’s Cramp Rating Scale (WCRS; severity based composite score where higher scores indicate worse posturing),^33^ Tubiana Musician’s Dystonia Scale (TMDS; 0–5 scale of musical performance ability where higher scores indicate better performance),^34^ the Michigan Hand Outcome Questionnaire (MHOQ; 0–100 score of patient reported hand function and ability),^35,36^ and a self-reported global impression of improvement measure [Clinical Global Impression-Improvement (CGI-I); a seven-point patient reported worsening/improvement Likert scale]. Health-related quality of life and non-motor symptoms were assessed using the Short Form-36,^37,38^ Beck Depression Inventory,^39^ and Mini Mental State Examination.^40^

Adverse events were systematically recorded using a standardized assessment template at each follow up timepoint, in addition to 1 day following the procedure as an additional safety measure. Objective measures of motor performance included assessments of strength, coordination and dexterity using grip and pincer strength tests, the Purdue 9-hole peg test,^41^ and the grooved pegboard test.^42^

### Neurophysiological recordings

#### Recording setup

EMG recordings were taken from abductor pollicis brevis (APB) using Ag-Ag surface electrodes with patients seated at rest. The EMG signals were amplified using a Digitimer D360 amplifier, band pass filtered 3–2000 Hz, analogue to digital converted at a sample rate of 5000 Hz and collected on a computer. TMS was then applied over the scalp using a 70-mm, hand-held figure-of-eight coil and Magstim BiStim pulse generator. The position of the hand area motor hotspot over the scalp was marked on a patient-specific cap used for each recording, with further manual refinement of optimal TMS coil positioning during each experiment. For median nerve stimulation, electrical stimulation was applied to the median nerve at the wrist using a constant current generator.

#### Motor threshold and motor-evoked potential recordings

After localization and marking for the APB and first dorsal interosseous (FDI) muscles, the resting motor threshold (RMT) was determined as the minimum stimulus to produce a 50-μV motor-evoked potential (MEP) in fie out of 10 consecutive trials. Active motor threshold (AMT) was determined during 20% maximum voluntary contraction of the APB and defined as the lowest stimulus intensity at which at least five MEPs of 150 μV amplitude were elicited in 10 consecutive trials.

The MEP amplitude was recorded over 30 trials with the stimulus intensity adjusted to produce an MEP amplitude of 1 mV. This intensity was then used throughout the experiment. Trials with any ongoing EMG artefact were rejected on-line.

#### Paired associative stimulation—conditioning protocol

Median nerve stimulation was applied at the wrist at 300% of perceptual threshold in the median distribution, stimulus duration 0.2 ms, delivered 25 ms before TMS over the APB at the intensity predetermined to yield a 1-mV resting MEP. Two hundred paired stimuli were delivered at a rate of 0.25 Hz. During PAS, patients were instructed to concentrate on the APB muscle and silently count the stimuli felt in the thumb. APB RMT, AMT, and MEPs were recorded before and 0, 15, and 30 min after PAS.^20^

#### Stimulus response curves

Stimulus response curves (SRCs) from the APB muscle were recorded with 5 trials in 9 steps of intensity from 10% to 90% TMS output. These were performed pre- and post-PAS at all timepoints.

#### Spatial specificity

Spatial specificity was analysed by comparing MEPs recorded from the FDI and APB following PAS.^14^ The FDI-APB MEP amplitude difference was used as a measure of spatial spread, and a descriptive normalized index was used to quantify the proportion of the baseline abnormality, relative to healthy controls, that was reduced following thalamotomy.

### Statistical analysis

All statistical analyses were performed in R (version 4.5.1; R Core Team, 2025) within the RStudio integrated development environment (version 2025.05.01 + 513; Posit Software, PBC). Figures were generated with GraphPad Prism (version 10.6.1 for macOS, GraphPad Software, Boston, MA, USA). Continuous variables are reported as mean ± standard deviation (SD) or median [interquartile range (IQR)], as appropriate. All statistical tests were two-tailed with statistical significance set at *P* < 0.05. For the mixed-effects models of the primary outcomes, *P*-values were obtained using parametric bootstrap method implemented via the *pbkrtest* package.^43^ Ninety-five percent confidence intervals (95% CI) were derived from model-based estimates of marginal means.

Longitudinal analyses were conducted using mixed-effects modelling to account for repeated measures within participants and inter-individual variability. Missing data were handled using maximum likelihood estimation within the mixed-effects modelling framework. Assumptions of the mixed-effects models were evaluated through visual inspection of residuals and formal diagnostics using the DHARMa package.

#### Clinical outcomes

Baseline characteristics were compared between intervention participants and healthy controls using independent samples *t*-tests for normally distributed variables and Wilcoxon rank-sum tests for non-normally distributed variables. Normality of baseline variables was assessed using the Shapiro–Wilk test.

Longitudinal changes were analysed using generalized linear mixed-effects models fitted with the *lme4* and *glmmTMB* packages. For primary clinical outcomes, models included fixed effects for treatment phase (pre- versus post-intervention) and time (in months) since intervention, with time modelled as a continuous variable to capture post-treatment trajectory. Random intercepts for participants were included to account for repeated measures and random slopes for participants over treatment were incorporated. Estimated marginal means and contrasts were derived using the *emmeans* package. Model robustness for primary clinical outcomes was assessed using parametric bootstrapping with 1000 simulations to estimate *P* values and confidence intervals using the *pbkrtest* package. Secondary clinical outcomes were analysed exploratorily with point estimates and confidence intervals alone reported.

#### Neurophysiological outcomes

Neurophysiological data were analysed offline using the Signal sweep-based data acquisition and analysis system (version 7.07; Cambridge Electronic Design, Cambridge, UK). For each trial, peak-to-peak MEP amplitudes were extracted and averaged within participants for each condition and timepoint prior to statistical analysis. All neurophysiological statistical analyses were subsequently performed in R.

To assess changes in cortical excitability, inhibition and associative plasticity, RMT, AMT, and MEP amplitudes were each modelled using linear mixed-effects models with fixed effects for PAS condition (pre-PAS and 0, 15 and 30 min post-PAS), timepoint (baseline, 1, 3, and 12 months), and their interaction. Random intercepts for participants were included to account for repeated measures.

Spatial specificity of PAS induced plasticity was examined by comparing MEP amplitudes recorded from the APB and FDI muscles across PAS conditions and timepoints using linear mixed-effects models. Estimated marginal means were extracted to calculate the FDI-APB difference at each timepoint, and a normalization index was derived to quantify convergence of intervention participants towards healthy control values.

SRCs were analysed using a mixed-effects slope modelling approach. MEP amplitude was modelled as the dependent variable, with stimulation intensity, PAS condition (pre- versus post-PAS), timepoint and their interactions included as fixed effects, and a random intercept for participant. This approach estimated the slope of the intensity–MEP relationship under each condition and timepoint. *Post-hoc* contrasts of estimated marginal trends were used to compare SRC slopes between pre- and post-PAS conditions within each timepoint.

Comparisons with healthy control participants were performed using group by condition interaction terms within the mixed-effects models.

## Results

### Study participants

Baseline trial participants are outlined in Supplementary Table 1. The mean age at the time of surgery was 51.27 ± 13.68 years (*range* 30–75), and average symptom duration was 16.50 ± 12.93 years (*range* 2–40). Eight participants were male and three were female. Of the total cohort of eleven participants, seven had writer’s cramp and six had musician’s dystonia. There were two participants who evidenced both writer’s cramp and musician’s dystonia. Those with musician’s dystonia played the violin (*n* = 2), the cello (*n* = 1), percussion/drums (*n* = 1), classical guitar (*n* = 1), and the recorder (*n* = 1). One participant was withdrawn from the study following the 3 month timepoint due to non-trial related issues. A cohort of 10 age (50.80 ± 15.07 years) and sex (seven male and three female) matched controls were recruited for comparison.

At baseline, there were no significant differences in either age or strength measures between cohorts (see Supplementary Table 2). For measures of dexterity, individuals with dystonia demonstrated slower times on the 9-hole peg test, though this did not differ significantly. The grooved pegboard test did, however, take significantly longer to perform in the dystonia cohort (103.00 s [90.9–113.7] versus 82.01 s [78.00–88.90], *P* < 0.01).

### Treatment parameters

The Vo-complex was targeted in all participants using traditional stereotactic planning. Lesions were made, on average, 12.36 ± 0.62 mm lateral to the midline, 12.06 ± 1.29 mm anterior to the posterior commissure and slightly above the AC-PC plane (0.09 ± 1.09 mm). On average, each participant received 10 ± 2.24 sonications with 3.00 ± 1.26 sonications above a therapeutic temperature (in excess of, or equal to, 54°C). Eight participants required more than 1 target lesion site within the Vo-complex based on intra-procedural response (*mean* 2.18 ± 0.75). The average lesion volume across the cohort was 150.36 ± 85.82 mm^3^ calculated on 1 month post-procedure scans (see Supplementary Table 3 for patient-specific lesion and sonication details; example day 1 and 12-month lesion in Supplementary Fig. 1).

In exploratory analyses, lesion volume was not significantly associated with changes in ADDS scores from baseline to 12 months. The Pearson correlation similarly showed a weak and imprecise linear relationship (*r* = 0.27, 95% CI: −0.39 to 0.75, *P* = 0.43). There was no association observed between a change in ADDS from baseline to 12 months and lesion location along the AP axis (ρ = 0.11, 95% CI: −0.67 to 0.81, *P* = 0.75) or the dorsoventral axis (ρ = 0.06, 95% CI: −0.58 to 0.81, *P* = 0.87). A moderate inverse association was observed along the mediolateral axis (ρ = −0.55, 95% CI: −0.93 to 0.23, *P* = 0.08), indicating uncertainty as to whether more medial lesions were associated with a better outcome.

### Ventro-oral-complex lesioning was associated with improvement in the symptoms of focal hand dystonia

#### Arm dystonia disability scale

ADDS scores improved immediately following treatment and this finding was sustained over 12-month follow-up. Estimated marginal means revealed that, at baseline, the mean ADDS score across the cohort was 47.14 (95% CI: 33.80 to 60.49). Following treatment, scores increased to 66.11 at 1 month (95% CI: 51.14 to 81.07), 65.96 at 3 months (95% CI: 51.42 to 80.51), 65.74 at 6 months (95% CI: 51.33 to 80.16), and 65.31 at 12 months (95% CI: 49.40 to 81.22). Linear mixed-effect modelling indicated a significant treatment effect (β = 19.04, 95% CI: 6.12 to 32.10, *P* < 0.01), confirmed using parametric bootstrap testing (*P* < 0.01). There was no significant reduction in scores over 12-month follow-up post-treatment (β = −0.07 per month, 95% CI: −0.88 to 0.74, *P* = 0.88; Fig. 1A).

**Figure 1:**
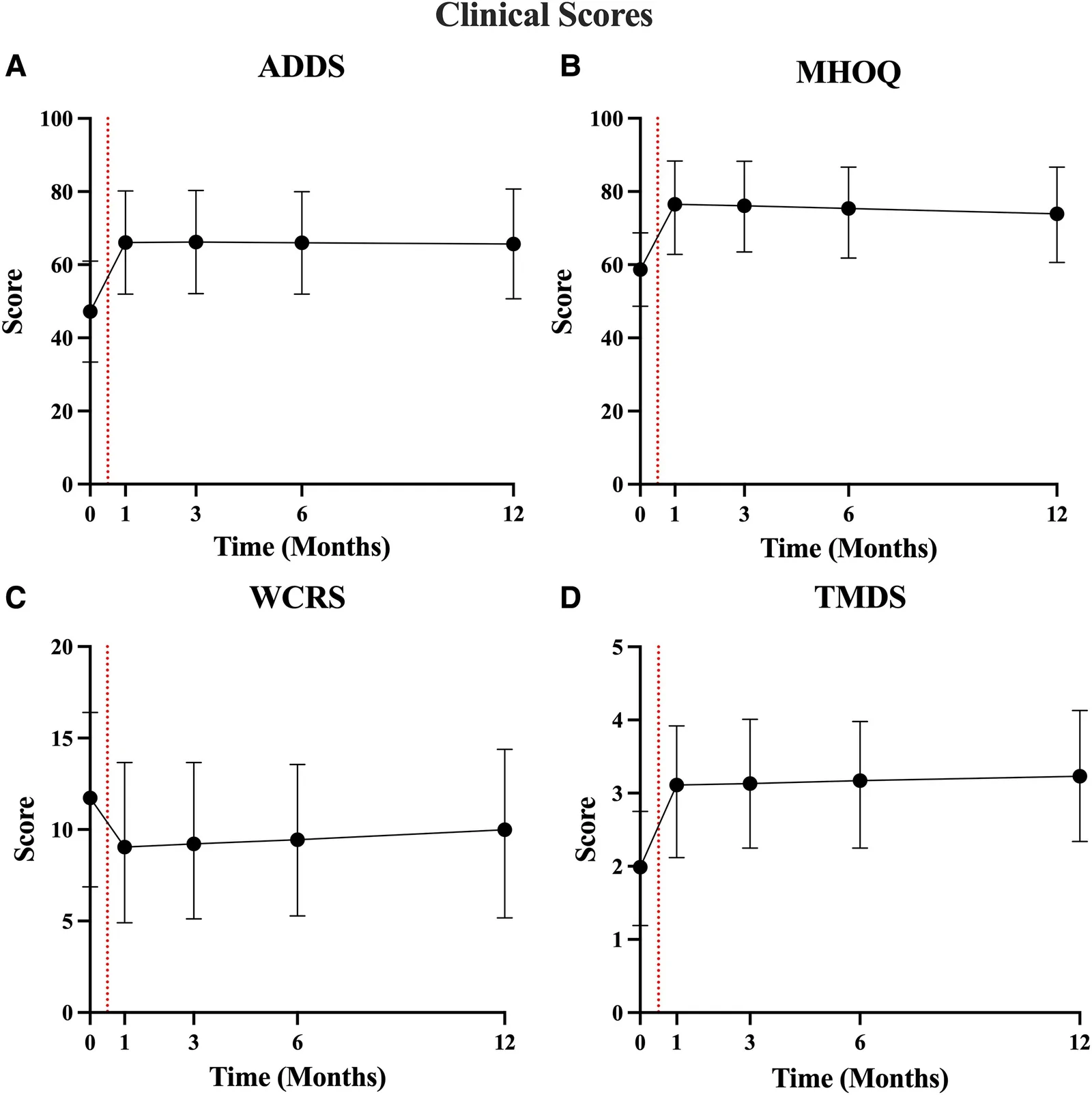
**Longitudinal clinical outcomes following HiFU Vo-complex thalamotomy.** Estimated marginal mean scores derived from linear mixed-effects models are shown for (**A**) Arm dystonia disability scale (ADDS), (**B**) Michigan hand outcomes questionnaire (MHOQ), (**C**) Writer’s cramp rating scale (WCRS), and (**D**) Tubiana musician’s dystonia scale (TMDS). Each data point represented the model-estimated marginal mean at the corresponding timepoint, shown at baseline (*n* = 11), 1-month (*n* = 11), 3-month (*n* = 10), 6-month (*n* = 10), and 12-month (*n* = 10) follow-up. Error bars represent the SEM. The dashed red line represents the time of Vo-complex thalamotomy. Higher scores represent an improvement in the ADDS, MHOQ, and TMDS, whilst lower scores represent improvement on the WCRS. Linear mixed-effects modelling demonstrated significant treatment effects for the ADDS (β = 19.04, *P* < 0.01), MHOQ (β = 18.00, *P* < 0.01), and TMDS (β = 1.10, *P* < 0.001), whereas the treatment effect for the WCRS was not significant (β = −2.39, *P* = 0.10).

#### Michigan hand outcomes questionnaire

Similar to the ADDS, MHOQ scores increased following treatment with a small, non-significant decline over 12 months. Estimated marginal means revealed that the mean MHOQ score at baseline was 58.66 (95% CI: 49.08 to 68.68). Following treatment, scores increased to 76.49 at 1 month (95% CI: 62.84 to 88.36), 76.13 at 3 months (95% CI: 63.49 to 88.29), 75.40 at 6 months (95% CI: 61.81 to 86.64), and 73.94 at 12 months (95% CI: 60.65 to 86.66). Linear mixed-effect modelling indicated a significant treatment effect (β = 18.00, 95% CI: 13.86 to 21.64, *P* < 0.01), confirmed using parametric bootstrap testing (*P* = 0.01). There was a subtle trend of small subsequent decline in scores over 12-month follow-up (β = −0.24 per month, 95% CI: −0.48 to 0.01, *P* = 0.09; Fig. 1B); however, this was not significant.

#### Writer’s cramp rating scale

WCRS scores reduced following treatment but did not reach pre-specified significance thresholds. At baseline, the mean WCRS score was 11.73 (95% CI: 6.87 to 16.40). Following treatment, scores reduced to 9.04 at 1 month (95% CI: 4.90 to 13.67), 9.22 at 3 months (95% CI: 5.12 to 13.66), 9.45 at 6 months (95%: 5.28 to 13.56) and 9.99 at 12 months (95% CI: 5.18 to 14.38). Linear mixed-effects modelling estimated a non-significant treatment effect of β = −2.39 (95% CI: −5.24 to 0.05, *P* = 0.10), confirmed on parametric bootstrap testing (*P* = 0.12). Following treatment, there was a small, non-significant upward trend in observed scores over 12 months (β = 0.08 per month, 95% CI: −0.28 to 0.48, *P* = 0.54; Fig. 1C).

#### Tubiana musician’s dystonia scale

TMDS scores demonstrated significant improvement following treatment. The mean baseline score was 1.99 (95% CI: 1.19 to 2.75). Post-treatment, the scores increased to 3.11 at 1 month (95% CI: 2.12 to 3.92), 3.13 at 3 months (95% CI: 2.25 to 4.01), 3.17 at 6 months (95% CI: 2.25 to 3.98) and 3.23 at 12 months (95% CI: 2.34 to 4.13). Linear mixed-effects modelling indicated a significant treatment effect (β = 1.10, CI: 0.90–1.32, *P* < 0.001), supported by parametric bootstrapping (*P* < 0.01). There was no evidence of decline in TMDS scores over time (β = 0.01 per month, 95% CI: −0.00 to 0.03, *P* = 0.62; Fig. 1D).

#### Global impression of improvement

On the CGI-I scale, most participants reported improvements following the procedure (Fig. 2). At day 1, 10/11 participants (91%) rated themselves as either ‘very much improved’ (18%) or ‘much improved’ (73%), with only one participant reporting no change. Over time, the proportion reporting significant improvement decreased modestly, with 7/11 participants (64%) feeling either ‘very much improved’ or ‘much improved’ at 1 month, 4/10 (40%) at 3 months, and 6/10 (60%) at 6 and 12 months. 4/10 (40%) participants reported ‘minimal improvement’ or ‘no change’ at 12 months with 1/10 (10%) reporting ‘much worse’ reflecting either treatment related side-effect or disease progression.

**Figure 2:**
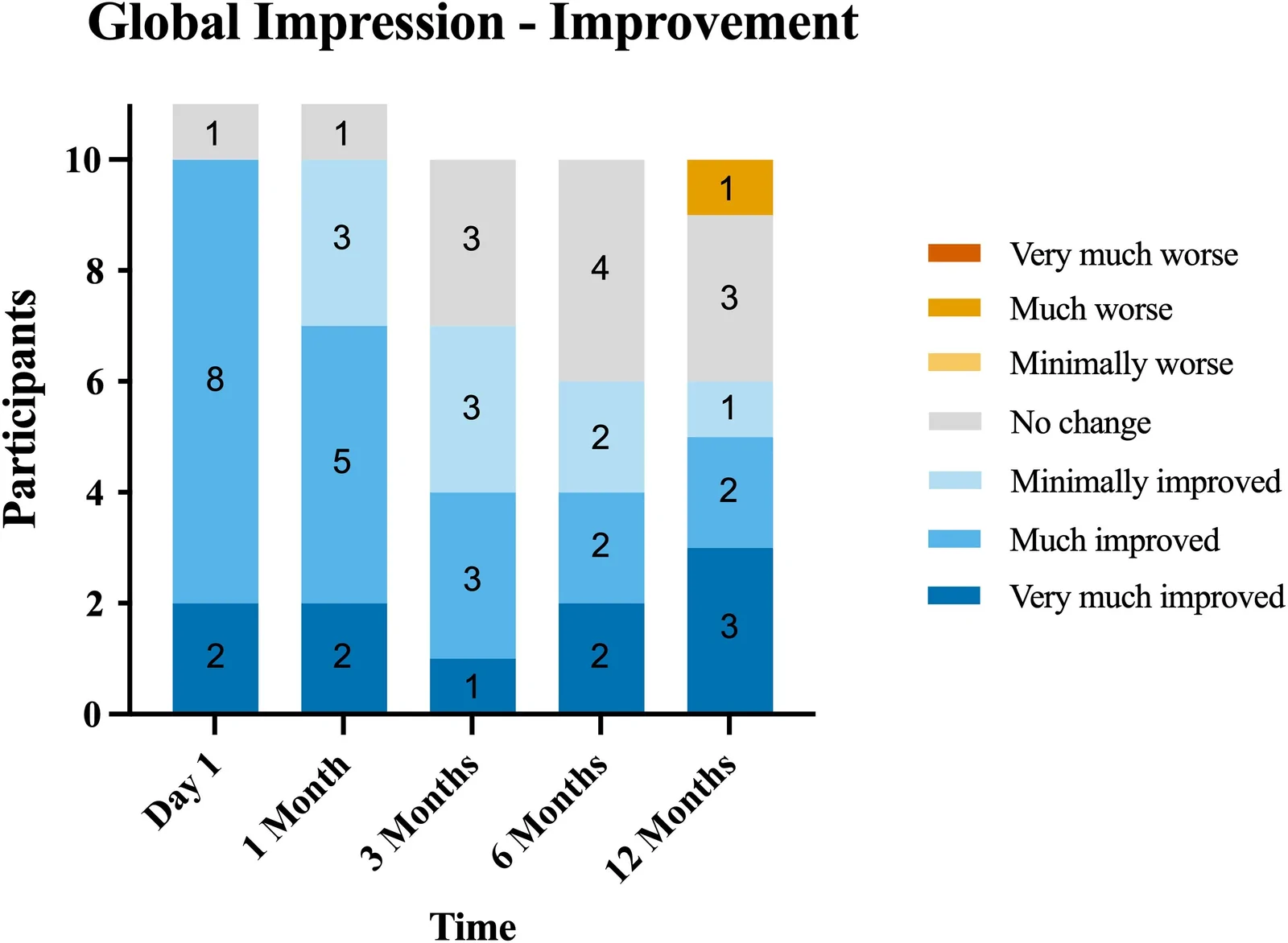
**Tracked global impression of improvement (CGI-I) following treatment.** Distribution of patient-reported perception of dystonia symptoms following Vo-complex thalamotomy. Stacked bars represent the number of participants in each response at each timepoint.

### No evidence that ventro-oral-complex lesioning was associated with permanent weakness or reduction in dexterity

Grip and pincer strength showed no clinically significant deterioration following Vo-complex thalamotomy (Supplementary Fig. 2A). Grip strength slightly decreased from 32.66 kg at baseline (95% CI: 24.48 to 40.52) to 29.74 kg at 1 month (95% CI: 20.17 to 39.71) and later increased to 35.04 kg at 12 months (95% CI: 21.01 to 40.29). Linear mixed-effects modelling showed no significant treatment effect (β = −3.54, 95% CI: −9.00 to 2.77, *P* = 0.27) but demonstrated a positive longitudinal trend over follow-up (β = 0.50 kg per month, 95% CI: 0.25 to 0.75, *P* < 0.001). Pincer strength increased slightly from 6.37 kg (95% CI: 5.12 to 7.75) to 7.10 kg at 1 month (95% CI: 5.85 to 8.31) before returning to baseline levels by 12 months (5.98 kg, 95% CI: 4.66 to 7.31). There was no significant treatment effect (β = 0.82, 95% CI −0.02 to 1.67, *P* = 0.06), with a small decline over time (β = −0.10 kg per month, 95% CI, −0.23 to 0.02, *P* < 0.001).

Measures of hand coordination and dexterity were similarly stable (Supplementary Fig. 2B). Time taken to complete the Purdue 9-hole peg test did not differ from baseline with an estimated treatment effect of β = 1.60 s (95% CI: −4.93 to 8.13, *P* = 0.63) and no change over 12 months (β = −0.084 s per month, 95% CI: −0.72 to 0.55, *P* = 0.79). The grooved pegboard test showed a non-significant increase in time taken to complete, being skewed by one outlier (β = 53.70 s, 95% CI: −38.70 to 164, *P* = 0.32), with no change over 12 months (β = −1.38 s, 95% CI: −8.80 to 5.94 *P* = 0.69).

### No evidence that ventro-oral-complex lesioning is associated with significant alterations in cognitive, mood, or general quality-of-life scores

Global cognitive performance, as assessed by the Mini Mental State Examination, remained stable following Vo-complex thalamotomy. Mean Mini Mental State Examination scores were 29.92 at baseline (95% CI: 29.58 to 30.00). There was no evidence of post-treatment decline in cognition (β = −0.25, 95% CI: −0.69 to 0.19, *P* = 0.30) and no change over follow-up (β = −0.013 per month, 95% CI: −0.06 to 0.03, *P* = 0.54).

Depressive symptoms, measured using the Beck Depression Inventory, increased slightly at early follow up but did not differ significantly from baseline. Scores increased from 3.06 at baseline (95% CI: 0.38 to 5.46) to 5.48 at 1 month (95% CI: 2.10 to 8.65), before gradually reducing to 4.18 at 12 months (95% CI: 0.51 to 8.14). There was no significant effect of treatment (β = 2.53, 95% CI −1.69 to 6.77, *P* = 0.26) and no change over follow-up (β = −0.12 per month, 95% CI −0.66 to 0.35, *P* = 0.60).

Health-related quality of life, assessed using the Short Form-36, was generally high at baseline across all domains and remained stable following treatment. Estimated treatment effects were small and non-significant (Supplementary Table 4).

### The safety profile of high-intensity focused ultrasound ventro-oral-complex thalamotomy is broadly comparable with that of existing ventral intermediate nucleus thalamotomy data

Both intra- and post-procedural adverse events were common (Table 1). Intraprocedural adverse events were observed in five participants (45%). All cases were considered mild (grade 1), and specific symptoms included vertigo, nausea, and head discomfort, relating to either the frame or to sonication intensity. Post-procedural adverse events were most frequently present during early follow-up, with seven participants (64%) reporting at least one adverse event at the 1-week and 1-month timepoints. The incidence of adverse events steadily declined over time, with three participants (30%) reporting symptoms at 3 months and two participants (20%) at 6 and 12 months. The majority of adverse events were mild (grade 1). Moderate adverse events (grade 2) were observed less frequently, while severe adverse events (grade 3) were limited to the early post procedural period and included weakness of the treated hemibody and fatigue/cognitive symptoms in two participants. One participant reported an acute worsening of mood related to comorbid medical issues during the study period.

**Table 1:** Adverse events both during and following HiFUS Vo-complex thalamotomy

Adverse event	Intraprocedural	Day 1	1 week	1 month	3 months	6 months	12 months
	*n* = 11	*n* = 11	*n* = 11	*n* = 11	*n* = 10	*n* = 10	*n* = 10
Patients	5 (45%)	5 (45%)	7 (64%)	7 (64%)	3 (30%)	2 (20%)	2 (20%)
*VIM comparison* ^22^	*76%*	*73%*	*78%*	*65%*	*37%*	*27%*	*28%*
Total number	8	10	17	12	6	2	4
Grade 1	8 (100%)	4 (40%)	10 (59%)	10 (83%)	5 (83%)	2 (100%)	4 (100%)
Grade 2	0 (0%)	4 (40%)	4 (24%)	2 (17%)	1 (17%)	0 (0%)	0 (0%)
Grade 3	0 (0%)	2 (20%)	3 (17%)	0 (0%)	0 (0%)	0 (0%)	0 (0%)
Gait		2	5	2	1	0	0
Dysmetria		0	0	0	0	0	0
Sensory		0	0	1	0	0	0
Dysarthria		2	4	1	0	0	0
Dysphagia		0	0	0	0	0	1
Fatigue/cognitive		2	3	3	2	0	1
Taste		1	1	1	0	0	0
Weakness		1	2	3	3	2	1
Headache		1	1	0	0	0	0
Vertigo		1	1	0	0	0	0
Other		0	0	1	0	0	1
Intraprocedural							
Vertigo	4						
Nausea	1						
Pain/headache	3						
Other	0						

At 12-month follow-up, eight participants (80%) reported no adverse effects. Two participants (20%) reported persistent weakness or fatigue/cognitive symptoms. Following study completion, one of these participants underwent neuropsychological assessment which identified subtle deficits in verbal memory.

Overall, the frequency and severity of adverse events in this cohort are consistent with published data on thalamic lesioning for movement disorders,^22^ although the pattern of symptoms differed. In contrast to tremor cohorts undergoing VIM ablation, where gait disturbance and sensory symptoms predominate, our cohort reported a greater proportion of transient weakness and cognitive complaints.

### Ventro-oral-complex thalamotomy attenuates paired associative stimulation–induced modulation of motor thresholds

Pre-PAS RMT and AMTs remained stable following Vo-complex thalamotomy, with no change evident at 1, 3, or 12 months compared with baseline (all *P* > 0.05; Supplementary Table 5).

PAS-modulated motor thresholds at baseline in individuals with focal dystonia, an effect that was attenuated following Vo-complex thalamotomy (Supplementary Table 6). At baseline, APB RMTs evidenced significant reductions following PAS at both 15- and 30-min assessment timepoints. Following thalamotomy, PAS no longer produced a reduction in RMT at either 1- or 3-month timepoints. At 12 months, PAS-related reductions in RMT recurred, resembling the pattern seen at baseline.

A similar, but weaker, pattern was observed with AMTs. At baseline, modest reductions in the APB AMTs were evident following PAS, although these did not reach significance. After thalamotomy, the magnitude of PAS-related AMT reduction was diminished; however, these changes did not meet pre-specified thresholds at any timepoint. There was no difference in the APB RMT and AMTs of individuals with focal dystonia and healthy control participants.

### Ventro-oral-complex thalamotomy reduces paired associative stimulation–induced motor cortex plasticity

The effect of PAS on MEP amplitudes of both the APB and FDI muscles was first examined at baseline in individuals with focal dystonia and healthy controls. In the APB, individuals with focal dystonia demonstrated a non-significant increase of 0.50 mV following PAS from 0.74 ± 0.08 to 1.24 ± 0.26 mV (mean increase 0.50 mV, 95% CI −0.35 to 1.35, *P* = 0.24, Fig. 3). Healthy controls exhibited a smaller non-significant increase of 0.24 mV from 0.69 ± 0.08 to 1.08 ± 0.13 mV (mean increase 0.39 mV, 95% CI −0.50 to 1.28, *P* = 0.38, Fig. 3). There was no significant interaction between the group and PAS condition, indicating comparable PAS-induced facilitation in the APB across groups at baseline.

**Figure 3:**
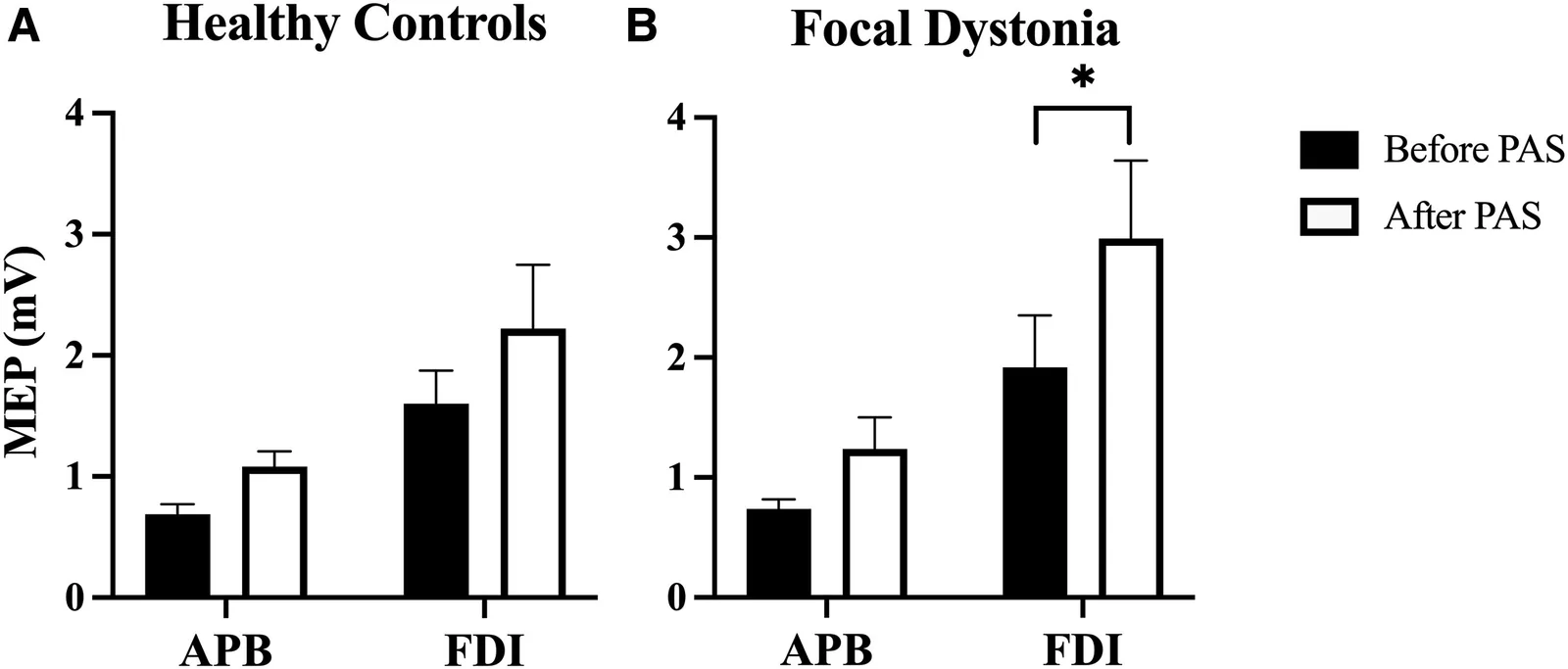
**Effect of paired associative stimulation (PAS) on abductor pollicis brevis (APB) and first dorsal interosseous (FDI) motor-evoked potentials (MEPs) in both healthy controls (A; *n* = 11) and individuals with focal dystonia (B; *n* = 10) at baseline.** Estimated marginal mean MEP amplitudes derived from linear mixed-effects models are shown before (pre-PAS) and after PAS (post-PAS). The bar charts represent the peak-to-peak amplitude of the MEPs recorded at baseline and are prior to intervention in the individuals with focal dystonia. The intensity of stimulation administered was that to obtain a 1 mV MEP in the APB muscle. The grey shaded bars represent the pre-PAS amplitude whilst the black bars represent the post-PAS amplitudes. Error bars represent the SEM (* = *P* < 0.05).

In contrast, PAS-related changes were also observed in the FDI, despite stimulation being restricted to the median nerve. Individuals with focal dystonia demonstrated a significant increase in FDI MEP amplitude from 1.92 ± 0.43 to 2.99 ± 0.65 mV (mean increase 1.07 mV, 95% CI 0.22 to 1.92, *P* = 0.01, Fig. 3). Healthy controls showed a smaller, non-significant mean increase from 1.60 ± 0.28 to 2.22 ± 0.53 mV (mean increase 0.62 mV, 95% CI −0.27 to 1.51, *P* = 0.17, Fig. 3). The presence of PAS-induced facilitation in a non-median nerve innervated muscle in individuals with focal dystonia suggests broader motor cortex excitability at baseline, with loss of spatial specificity.

To assess the effect of Vo-complex thalamotomy on PAS induced corticospinal facilitation over time, linear mixed-effects modelling was performed with fixed effects for timepoint, PAS condition and their interaction, and a random intercept for subject. When baseline data were incorporated into the longitudinal model, PAS significantly facilitated corticospinal excitability at baseline in both muscles. In the APB, PAS was associated with an average increase of 0.59 mV (95% CI 0.05 to 1.14, *P* = 0.03), while in the FDI, the average facilitation was 1.11 mV (95% CI 0.30 to 1.92, *P* = 0.01). These effects were attenuated following Vo-complex thalamotomy, with PAS related increases in both APB and FDI MEP amplitude no longer reaching statistical significance at 1-, 3-, or 12- month follow-up (Table 2 and Supplementary Fig. 3).

**Table 2:** Paired associative stimulation (PAS)-induced facilitation of abductor pollicis brevis (APB) and first dorsal interosseous (FDI) motor-evoked potential (MEP) amplitudes at baseline and after ventro-oral (Vo-)complex thalamotomy

APB	Pre	0 min	15 min	30 min	Avg facilitation	*P* (Avg post versus pre)
Baseline	0.74 ± 0.26	1.24 ± 0.26	1.34 ± 0.27	1.40 ± 0.27	+0.59	0.03*
1 Month	0.71 ± 0.28	1.14 ± 0.28	1.21 ± 0.30	1.10 ± 0.34	+0.44	0.15
3 Month	0.76 ± 0.32	0.82 ± 0.32	1.31 ± 0.32	1.08 ± 0.37	+0.31	0.37
12 Month	1.21 ± 0.27	1.30 ± 0.27	1.50 ± 0.27	1.61 ± 0.27	+0.26	0.36
FDI						
Baseline	1.92 ± 0.52	3.01 ± 0.52	2.96 ± 0.53	3.11 ± 0.53	+1.11	<0.01*
1 Month	1.37 ± 0.55	2.38 ± 0.55	2.18 ± 0.57	1.97 ± 0.62	+0.80	0.08
3 Month	1.96 ± 0.59	1.95 ± 0.59	2.26 ± 0.59	2.44 ± 0.66	+0.26	0.62
12 Month	2.24 ± 0.54	2.51 ± 0.54	2.55 ± 0.54	2.84 ± 0.54	+0.39	0.36

Estimated marginal mean MEP amplitudes (mV ± SEM) for the APB and FD) muscles are shown under all PA) conditions at baseline (*n* = 11), 1 (*n* = 11), 3 (*n* = 10), and 12 (*n* = 10) months following thalamotomy. The average (Avg) facilitation represents the mean post-PAS change relative to the pre-PAS value at that timepoint. *P*-values are derived from the comparison of the averaged post-PAS response and the pre-PAS response at each timepoint (* = *P*  *<* 0.05). All values are derived from linear mixed-effects models.

### Ventro-oral-complex thalamotomy reduces spatial spread of paired associative stimulation–induced corticospinal facilitation

Spatial specificity of PAS-induced corticospinal facilitation was assessed by comparing MEP amplitudes in the APB and FDI muscles across healthy controls and individuals with focal dystonia. Differences in MEP amplitudes between the FDI and APB (FDI-APB difference) were examined pre- and post-PAS at baseline and following Vo-complex thalamotomy.

In healthy control participants, FDI MEP amplitudes were consistently higher than APB MEP amplitudes under all PAS conditions, with a modest increase in the FDI-APB difference following PAS. In individuals with focal dystonia, the FDI-APB difference was larger at baseline compared with controls and was further accentuated following PAS, consistent with reduced spatial specificity of PAS-induced facilitation (Table 3).

**Table 3:** Baseline motor-evoked potential (MEP) amplitudes and spatial specificity in control and intervention participants

Group/condition	APB	FDI	FDI − APB	*P*	Group/condition	APB	FDI	FDI − APB	*P*
Controls/pre-PAS	0.69 ± 0.46	1.60 ± 0.46	0.91	0.08	Intervention/pre-PAS	0.74 ± 0.44	1.92 ± 0.44	1.18	0.02*
Controls/0 min	0.94 ± 0.46	2.00 ± 0.46	1.06	0.04*	Intervention/0 min	1.24 ± 0.44	3.01 ± 0.44	1.77	<0.001*
Controls/15 min	1.21 ± 0.46	2.21 ± 0.46	1.01	0.05	Intervention/15 min	1.25 ± 0.45	3.03 ± 0.45	1.79	0.001*
Controls/30 min	1.11 ± 0.46	2.45 ± 0.46	1.34	0.01*	Intervention/30 min	1.30 ± 0.45	3.18 ± 0.45	1.88	<0.001*

Estimated marginal mean MEP amplitudes (mV ± SEM) for the abductor pollicis brevis (APB) and the first dorsal interosseous (FDI) muscles shown under each PAS condition. Spatial specificity is expressed as the FDI-APB difference. Values derived from linear mixed-effects models in 11 participants. *P*-values represent within-group comparisons of FDI versus APB MEP amplitudes for each condition (* = *P*  *<* 0.05).

Following Vo-complex thalamotomy, there was a consistent reduction in FDI-APB difference under all PAS conditions, indicating attenuation of spatial spread, although these changes did not reach statistical significance. Post thalamotomy values demonstrated a directional shift towards those observed in healthy control participants (Table 4).

**Table 4:** Changes in spatial specificity following Vo-complex thalamotomy

Group/Condition	APB	FDI	FDI − APB	NI (%)	Δ (Post − Baseline)	*P* (versus baseline)	*P* (versus controls)
Intervention post-thalamotomy/pre-PAS	0.84 ± 0.28	1.76 ± 0.28	0.93	94	−0.26	0.43	0.98
Intervention post-thalamotomy/0 min	1.04 ± 0.27	2.21 ± 0.27	1.17	86	−0.61	0.06	0.84
Intervention post-thalamotomy/15 min	1.25 ± 0.28	2.29 ± 0.28	1.04	96	−0.64	0.06	0.95
Intervention post-thalamotomy/30 min	1.19 ± 0.29	2.52 ± 0.29	1.33	102	−0.56	0.11	0.99

Estimated marginal mean motor-evoked potential (MEP) amplitudes for the abductor pollicis brevis (APB) and first dorsal interosseous (FDI) shown post-thalamotomy across paired associative stimulation (PAS) conditions (*n* = 11). Spatial specificity was quantified as the FDI-APB MEP difference under each condition. The normalization index (NI) represents the proportion of shift in the FDI-APB difference towards the values observed in healthy control participants (*n* = 10) where a value of 100% indicates alignment with controls, 0% no change and >100% over-correction relative to control participants. Values are derived from linear mixed-effects models. Δ (Post − Baseline) represents the change in FDI-APB difference following thalamotomy. *P*-values (versus baseline) represent within-subject comparisons of post-thalamotomy and baseline FDI-APB differences, and *P*-values (versus control) represent comparisons of post-thalamotomy FDI-APB differences with control baseline values derived from linear mixed-models.

Using values averaged across the 1-, 3-, and 12-month timepoints, post PAS FDI-APB differences approached control values. To quantify this change, a descriptive normalization index (NI) of shift towards control values was calculated as:


$$\mathrm{NI}(\text{\%})=100\;*\;\frac{\left(\Delta \mathrm{Pr}e-\mathrm{thalamotomy}-\Delta P\mathrm{ost}-\mathrm{thalamotomy}\right)}{\left(\Delta \mathrm{Pr}e-\mathrm{thalamotomy}-\Delta \mathrm{Control}\right)}$$

where Δ is the FDI-APB amplitude difference (mV) for each PAS condition. An NI of 100% indicates alignment with control values, 0% indicates no change, and >100% indicate that post-thalamotomy values crossed the control mean.

Using this metric, the pre-PAS FDI-APB difference improved by 94% towards control values. Post-PAS responses demonstrated a similar pattern, with NI values of 85% immediately post-PAS, and 95% and 102% at 15 and 30 min post-PAS, respectively (Table 4).

### Ventro-oral-complex thalamotomy alters paired associative stimulation-induced modulation of stimulus response curves

The effects of Vo-complex thalamotomy on SRCs before and after PAS were examined using linear mixed-effects modelling. Across all timepoints, SRCs demonstrated the expected increase in MEP amplitude with increasing TMS intensity.

At baseline, PAS facilitated corticospinal excitability, with post-PAS SRCs exhibiting steeper slopes compared to pre-PAS (Fig. 4A). Linear mixed-effects modelling revealed a significant three way interaction between stimulation intensity, PAS condition, and timepoint (*F* = 7.59, *P* < 0.001). Estimated marginal trends demonstrated that, prior to thalamotomy, post-PAS slopes were significantly greater than pre-PAS (β = −0.01, *P* = 0.005).

**Figure 4:**
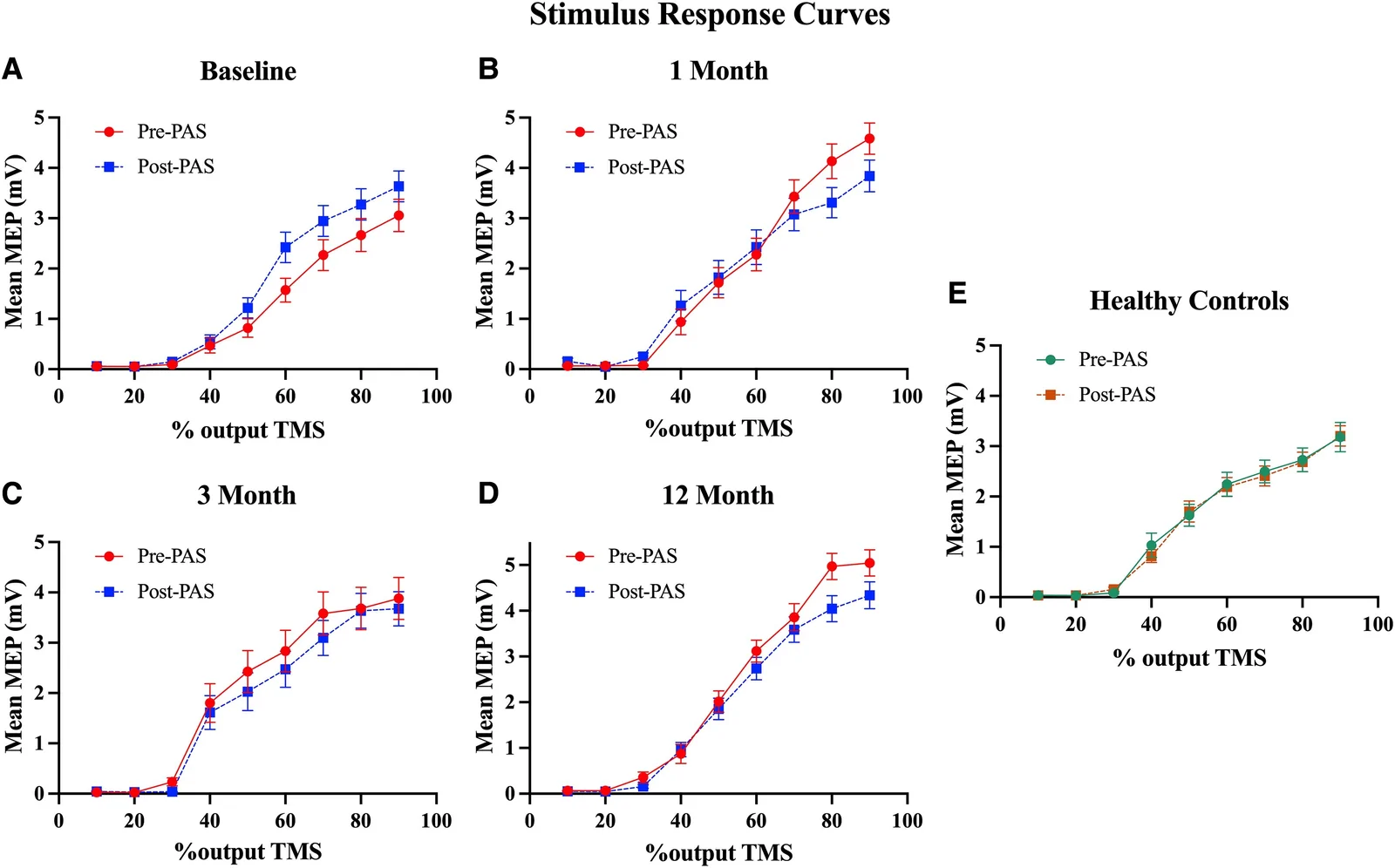
**Stimulus response curves (SRCs) at baseline and follow-up timepoints before and after paired associative stimulation (PAS).** Estimated marginal mean motor-evoked potential (MEP) amplitudes derived from linear mixed-effects models are plotted as a function of transcranial magnetic stimulation (TMS) intensity (%output) at four timepoints for the abductor pollicis brevis (APB) muscle before (pre-PAS) and after (post-PAS) at (**A**) baseline (*n* = 11), (**B**) 1 month (*n* = 11), (**C**) 3 months (*n* = 10) and (**D**) 12 months (*n* = 10) post ventro-oral (Vo-)complex thalamotomy. (**E**) Corresponding SRCs for healthy control participants (*n* = 10). Each data point represents the model-estimated marginal mean MEP amplitude at the corresponding stimulation intensity, and error bars represent the SEM. Linear mixed-effects modelling demonstrated a significant three-way interaction among stimulation intensity, PAS condition, and timepoint (F = 7.59, *P* < 0.001). Estimated marginal trend analyses showed significantly steeper post-PAS SRC slopes at baseline (*P* = 0.005), reversal of this effect at 1 month (*P* = 0.002) and 12 months (*P* = 0.006), and no significant difference at 3 months (*P* = 0.49). No significant pre- versus post-PAS difference was observed in healthy controls (*P* = 0.95).

Following Vo-complex thalamotomy, PAS no longer enhanced SRC slopes. At 1 month, pre-PAS slopes exceeded post-PAS slopes (β = 0.012, *P* = 0.002, Fig. 4B), indicating attenuation and reversal of PAS-induced facilitation. This pattern persisted at 12 months (β = 0.012, *P* = 0.006; Fig. 4D). At 3 months, there was no significant difference between pre- and post-PAS slopes (β = 0.003, *P* = 0.49; Fig. 4C).

In healthy controls, SRCs showed the expected increase in MEP amplitude with increasing TMS intensity, with no significant difference in slope or intercept between pre- and post-PAS states (β = −0.01, *P* = 0.95), indicating stable corticospinal excitability (Fig. 4E).

Model-predicted SRCs were consistent with the statistical contrasts and are presented in Supplementary Fig. 4.

## Discussion

We demonstrated that HiFu Vo-complex thalamotomy alters neuroplastic mechanisms, as measured by TMS, and results in symptom improvement in focal hand dystonia. While VIM and PSA HiFU lesioning has been extensively studied for the treatment of tremor,^21,22,44-46^ in addition to more recent evidence for PTT^47^ and STN lesioning^48^ in PD, the evidence for Vo-complex HiFU lesioning in focal dystonia remains limited,^23^ and the biological basis of any symptom improvement remains unexplored.

### Ventro-oral-complex thalamotomy modestly improves symptoms of focal hand dystonia

The observed improvements in the ADDS and TMDS are consistent that reported in a recent pilot study of HiFU Vo-complex thalamotomy for focal dystonia.^23^ Our findings of improvement in the MHOQ are novel and contribute to the evidence base for thalamic lesioning in dystonia. Unlike those findings reported by Horisawa *et al.*,^23^ we did not identify improvement in the WCRS in our study. This may be related to a greater severity of writer’s cramp in our cohort, evidenced by higher baseline scores (11.20 ± 4.60 versus 6.10 ± 2.90). The WCRS scores arm, wrist, and finger posturing during a writing task, however individuals with writer’s cramp often develop compensatory strategies to preserve handwriting quality. It may be that subtle abnormalities of fine motor control occur after lesioning that improve comfort, fatiguability and reduce reliance on these compensatory strategies, without altering gross posture, accounting for the disconnect in objective scores and subjective symptom improvement (see Supplementary Fig. 5 for a case example where the patient’s handwriting quality is markedly improved however the WCRS did not change).

Recurrence of focal dystonia following initial improvement after Vo-complex thalamotomy, whether performed with HiFU, radiofrequency ablation, or gamma-knife surgery, has been extensively reported. In a large retrospective cohort of 171 patients undergoing radiofrequency Vo-complex ablation, Horisawa *et al.*^25^ observed recurrence in 10% of cases, attributed to incomplete ablation of the target nucleus. In their recent HiFU study, recurrence occurred in 30% of participants (*n* = 3). Using the ADDS and MHOQ in our study, one participant experienced no improvement following treatment and a second participant experienced significant initial improvement at 1 month, followed by worsening to scores below baseline for the remainder of the trial period. Compared with our cohort, Horisawa *et al.*^23^ performed HiFU lesioning with larger total lesion volumes (328 ± 142 versus 150 ± 86 mm^3^, *P* < 0.01), with a trend towards a greater number of therapeutic sonications >54°C (4.1 ± 1.2 versus 3.0 ± 1.3, *P* = 0.08). SDRs and total sonication numbers were similar, suggesting that the larger lesions in the trial performed by Horisawa *et al.*^23^ may have contributed to their greater clinical benefit, however in exploratory analyses performed in our study, lesion volume was not correlated with improvement in ADDS scores. The exact lesion coordinates in their cohort^23^ were not published, however presuming that lesions were made at the described Vo-complex target, our lesioning was performed slightly more anterior and medial, with the aim of greater capsular clearance, with a non-significant trend towards more medial lesions being associated with improved clinical outcome. Recently, using voxel-wise probabilistic lesion mapping, Nishanti *et al.*^49^ identified that lesioning the border zone between the thalamic ventro-oral posterior and VIM nuclei was associated with the greatest improvement in focal hand dystonia, strengthening the argument for target refinement under future prospective controlled trial conditions.

Adverse events were common but consistent with rates reported by our centre for VIM thalamotomy in tremor.^22^ The side-effect profile differed, however, likely reflecting either the distinct functional topography of the thalamus targeted in this procedure, or disease related factors. Fatigue/cognitive disturbance and weakness were more common. Intraprocedural vertigo was noted in 4/11 participants, indicating that this phenomenon is not necessarily specific to the VIM.^50^ One participant reported issues with post-procedural tempo control, and one participant underwent formal neuropsychological testing with evidence of subtle deficits in verbal memory following left thalamic lesioning.

### Ventro-oral-complex thalamotomy modifies corticospinal excitability and reduces spatial spread potentially gate or ‘reset’ motor selection

Vo-complex HiFU thalamotomy was associated with improvement in the ADDS, MHOQ, and TMDS. One proposed mechanism, based on the evident neurophysiological changes, is that Vo-complex thalamotomy may modulate the cortico-basal ganglia-thalamo-cortical loop. A recent correspondence article in *Parkinsonism and Related Disorders*^12^ proposes that the correction of aberrant learned motor patterns likely requires permanent circuit or network modification, and therefore, improvement in symptoms should be associated with evidence of such a change. This study demonstrates altered neuroplastic responses as measured by TMS. Individuals with dystonia evidenced excessively heightened facilitation of the FDI, a non-median nerve innervated muscle, compared to healthy controls, when PAS was applied to the median nerve only. A trend towards control values, in addition to modification of post-PAS SRCs, evidences a direct effect of this procedure on PAS induced cortical excitability.

Previous literature has explored the breakdown of centre-surround inhibition within the motor circuit in individuals with focal dystonia.^51,52^ The result is unwanted activation of non-target muscles and the activation of abnormal motor patterns. Our findings of a loss of spatial specificity are in keeping with this and echo that reported by Quartarone *et al.*^16^ where MEP facilitation in both the APB and FDI exceed that noted in healthy control participants. Importantly, the response to PAS was significantly attenuated following Vo-complex thalamotomy, with reduced MEP facilitation, dampened RMT modulation and reduced spatial spread.

DBS of the GPi is an effective treatment for generalized dystonia.^53,54^ Reversible reductions in corticospinal excitability have been demonstrated in the DBS ON and OFF state.^20,55^ The GPi DBS ON state abolishes excitatory motor cortex plasticity in generalized dystonia with a rightward shift compared to the DBS OFF state. This effect was evident in our cohort where the SRCs, at baseline, demonstrated a leftward shift with increasing TMS intensities producing proportionately larger MEPs. Following thalamotomy, PAS no longer enhanced corticospinal excitability. Notably, while PAS-derived metrics appeared sensitive to group-level alterations in corticospinal plasticity, exploratory analysis did not demonstrate an association between change in PAS slope and clinical outcome, indicating that these measures alone do not account for treatment, response but rather may serve as a marker of alterations in motor pathway gating mechanisms.

### Limitations

Our sample size and the absence of a control or sham treatment group limit statistical inference of the relationship between neuroplastic change and symptom improvement. The small sample size increases type II error, particularly in the context of this rare condition. Placebo, expectation, and time-based disease progression are potential confounders.

MRI-guided neuronavigation was not used to determine the optimal TMS coil position during the neurophysiological assessments. We implemented a patient-specific landmark-notated cap with motor hotspot validation occurring at each TMS session to reduce the impact of coil-position variability on the longitudinal assessment of motor thresholds, MEP amplitudes and stimulus response curve analyses. This does not, however, provide the same accuracy and reproducibility of MRI-guided neuronavigation and may have impacted longitudinal neurophysiological follow-up.

The cohort that was studied was heterogenous, with varying expressions of focal hand dystonia and task specificity. It may be that there are subtle differences in disease duration, subtype and severity that influence clinical and neurophysiological responses. Although changes in PAS-induced facilitation were clear and identified, these act as surrogate markers for neuroplasticity, and there is conjecture in the literature as to whether abnormal plasticity forms a state or trait in individuals with dystonia. As we did not find a relationship between the change in PAS slope and clinical improvement, any extrapolation that altered plasticity will be reflected by symptom improvement should be cautioned.

## Conclusions

We have demonstrated that improvements in the symptoms of focal hand dystonia following HiFU Vo-complex thalamotomy are associated with identifiable changes in corticospinal excitability, neuroplastic gain and suggest reduced spatial spread. Separate unpublished resting state fMRI data in this cohort suggests that changes in network reorganization following Vo-complex thalamotomy are modest. Therefore, this treatment may in fact act as an interruption of pathologic pallidothalamic relay activity, sufficient to modulate downstream motor control, without resulting in large-scale network reorganization. This reset, or modulation, of motor control thereby restores the appropriate conditions for motor programme recoding and correction of task performance. While the improvement in the symptoms of focal hand dystonia were statistically significant, and the side effect profile acceptable, further randomized controlled trials that incorporate blinding are needed to determine whether Vo-complex HiFU ablation is an effective treatment in medically refractory focal hand dystonia.

Future research should be aimed at refining therapeutic targeting and identifying appropriate treatment volumes as well as neurophysiological and imaging biomarkers that may predict treatment response in an effort to broaden our understanding of the circuit dynamics that provoke abnormal motor patterns and sustain the symptoms of focal hand dystonia. Incorporation of formal pre- and post-neuropsychological testing may better elucidate subtle cognitive effects of Vo-lesioning. These directions provide an opportunity to build on the mechanistic insights that we have presented, with the aim of advancing personalized circuit-informed strategies for the treatment of focal hand dystonia and other movement disorders.

## Supplementary Material

fcag403_Supplementary_Data

## Data Availability

The R scripts used to perform the statistical analyses reported in this study are publicly available at: https://github.com/jmaamary/BrainCommunications_FHD_HiFU. De-identified participant data are available from the corresponding author upon reasonable request, subject to institutional ethics approval and data-sharing agreements.
