# Supplementary material for: Focused ultrasound ventro-oral thalamotomy alters cortical excitability and motor control in focal hand dystonia
Source: Brain Commun. 2026 Oct 23;8(6):fcag403. doi: 10.1093/braincomms/fcag403 (PMC13729118; doi:10.1093/braincomms/fcag403)
Supplement: Supplementary file 1 [file fcag403_supplementary_data.pdf]

Supplementary Table 1 Intervention group baseline characteristics

Age at procedure, y	51.27 ± 13.68
Sex, M/F	8 / 3
Disease duration, y	16.5 ± 12.93
Age at onset, y	35.25 ± 12.93
Writer's cramp	7
Musician's dystonia	6

Data are presented as means ± standard deviation

Supplementary Table 2 Intervention group baseline characteristics

	**Intervention (n = 11)**	**Control (n = 10)**	***P***
Age, y	51.27 ± 13.68	50.80 ± 15.07	0.94
Grip strength, kg	32.37 ± 14.90	37.38 ± 15.40	0.46
Pincer strength, kg	6.37 ± 2.27	6.46 ± 1.81	0.93
9-hole peg test, sec	21.90 [19.80-26.90]	19.50 [18.20-21.50]	0.10
Grooved pegboard test, sec	103.00 [90.90-113.70]	82.00 [78.00-88.90]	**< 0.01**

Data are presented as mean ± standard deviation for normally distributed variables and median [interquartile range] for non-normally distributed variables. Normality was assessed using the Shapiro–Wilk test. Between-group comparisons were performed using independent-samples *t*-tests for normally distributed variables and Wilcoxon rank-sum tests for non-normally distributed variables.

Supplementary Table 3 Patient specific sonication and lesion details

Case	SDR	Sonications (n)		Temperature (high)		Lesion location (normalised left hemisphere)			Lesion Volume
		Total	≥ 54°C	Average	Max	X	Y	Z	(mm^3)
1	0.30	9	4	56	61	-11.49	-10.90	0.26	222
2	0.30	9	2	56	58	-11.67	-13.48	1.48	33
3	0.50	11	2	56	59	-11.85	-12.35	2.03	68
4	0.50	11	3	56	60	-12.94	-10.16	-0.93	214
5	0.42	10	3	56	60	-11.51	-11.42	-0.15	193
6	0.43	13	5	55	59	-12.39	-10.62	-0.42	311
7	0.45	14	4	56	60	-13.03	-12.93	-0.66	180
8	0.46	11	2	57	60	-12.82	-13.22	-0.25	157
9	0.39	7	2	55	58	-12.70	-10.84	-1.10	105
10	0.33	8	2	54	56	-12.44	-13.08	-0.75	36
11	0.45	8	3	57	61	-13.10	-13.66	1.51	135
Avg (± SD)	0.41 ± 0.07	10 ± 2.24	3.00 ± 1.26	55.82 ± 0.87	59.27 ± 1.49	-12.36 ± 0.62	-12.06 ± 1.29	0.09 ± 1.09	150.36 ± 85.82

Data are presented as means ± standard deviation. Lesion coordinates are normalised for the left hemisphere in all participants.

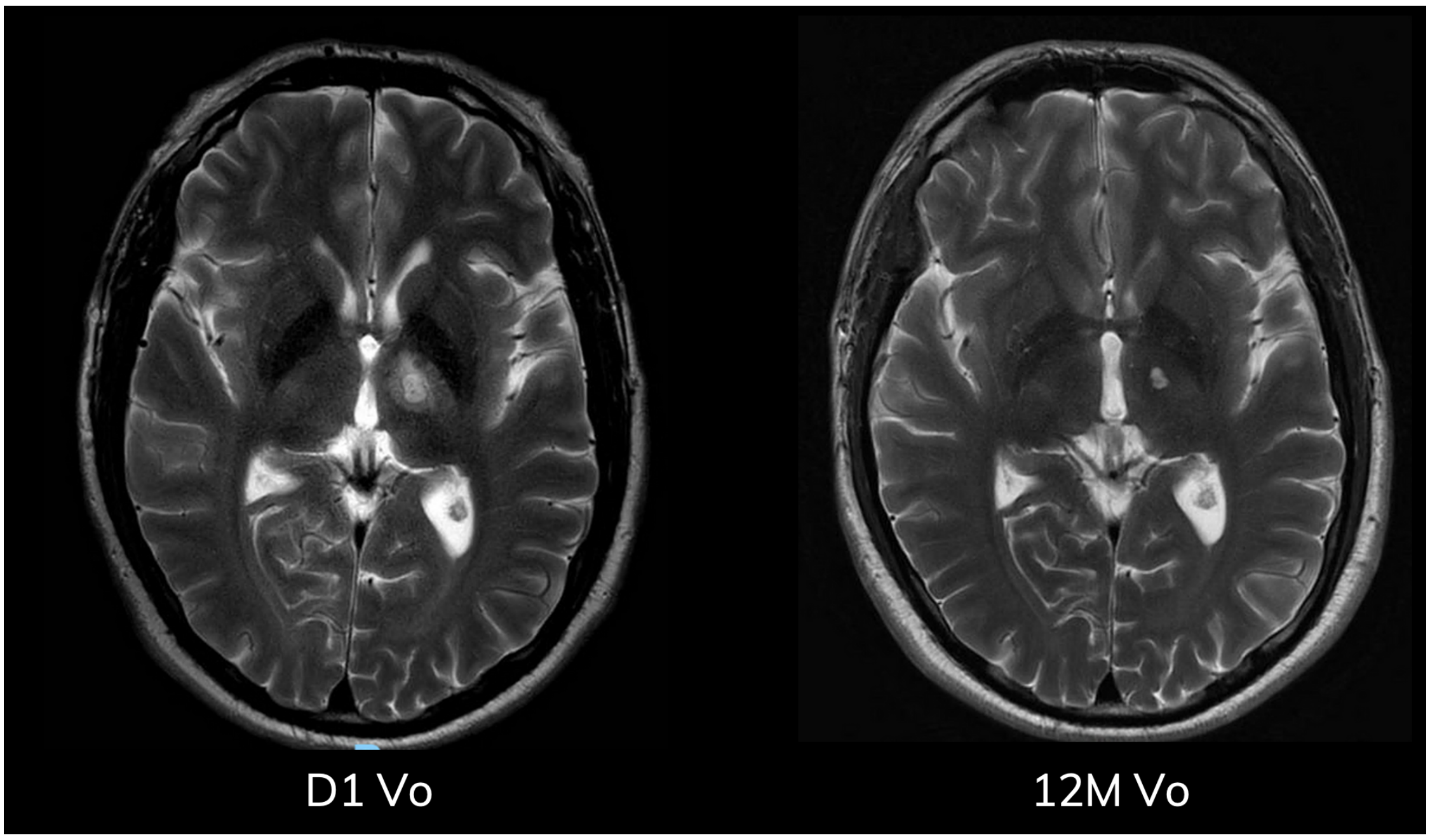

Supplementary Figure 1 Longitudinal evolution of the ventro-oral (Vo) thalamotomy lesion. Axial T2-weighted MRI obtained 1 day after treatment (left) demonstrates a well-defined Vo lesion with surrounding oedema. At 12 months (right), the lesion appears smaller with expected signal evolution and resolution of oedema.

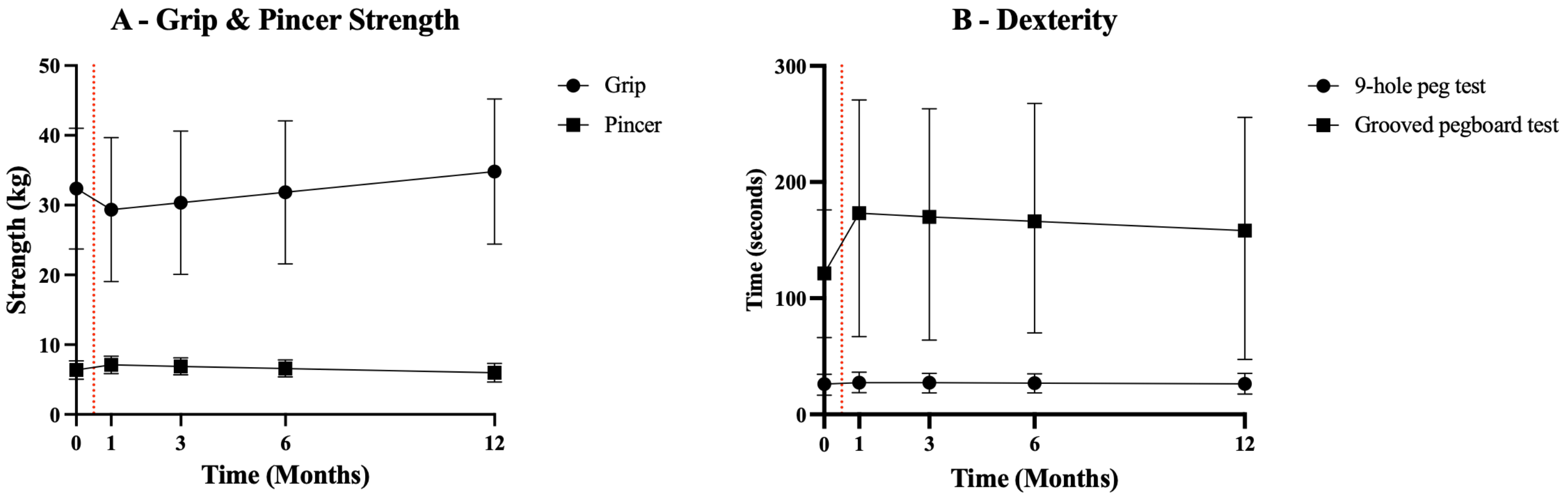

Supplementary Figure 2 Measures of motor function following Vo-complex thalamotomy. (A) Estimated marginal mean grip and pincer strength and **(B)** estimated marginal mean time to complete the Purdue 9-Hole Peg Test and Grooved Pegboard Test at baseline (n = 11), 1 month (n = 11), 3 months (n = 10), 6 months (n = 10), and 12 months (n = 10) following Vo-complex thalamotomy. Each data point represents the model-estimated marginal mean at the corresponding study timepoint. Error bars represent the standard error of the mean (SEM). The dashed red line indicates the time of Vo-complex thalamotomy. Treatment effects were estimated using linear mixed-effects models with fixed effects for timepoint and random intercepts for participant. Linear mixed-effects modelling demonstrated no significant treatment effect for grip strength (β = -3.54 kg, p = 0.27), pincer strength (β = 0.82 kg, p = 0.06), Purdue 9-Hole Peg Test (β = 1.60 s, p = 0.63), or Grooved Pegboard Test (β = 53.70 s, p = 0.32).

Supplementary Table 4 Short form-36 domain scores at baseline and following treatment

SF-36 Domain	Baseline (n = 11)	1 month (n = 11)	3 months (n = 10)	6 months (n = 10)	12 months (n = 10)	Treatment effect	*p-value*
**Physical functioning**	90.93 (82.86-98.45)	90.54 (83.35-97.46)	90.14 (83.78-97.14)	89.64 (82.71-95.61)	88.60 (80.42-96.98)	0.02 (-8.76- 9.17)	0.98
**Role physical**	80.40 (58.82-101.25)	58.67 (40.78-80.02)	60.63 (42.48-79.15)	63.66 (44.44-81.39)	68.61 (41.32-91.54)	-22.90 (-53.90-7.09)	0.13
**Role emotional**	81.59 (60.17-101.42)	88.80 (70.94-105.43)	86.38 (71.76-101.04)	83.08 (70.60-97.84)	76.30 (56.28-96.94)	8.45 (-20.30-35.80)	0.56
**Energy/Fatigue**	60.32 (47.75-73.89)	66.25 (54.86-76.71)	65.95 (55.82-74.84)	65.31 (56.06-74.35)	63.99 (50.76-77.20)	6.08 (-10.10-25.40)	0.46
**Emotional wellbeing**	79.47 (69.37-88.95)	80.18 (71.87-88.16)	79.86 (73.18-87.71)	79.40 (73.76-87.43)	78.60 (67.43-89.14)	0.29 (-13.70-14.00)	0.96
**Social functioning**	87.35 (77.90-100.42)	80.23 (66.90-93.77)	80.80 (68.06-93.77)	81.52 (70.01-94.27)	83.38 (69.40-99.93)	-7.02 (-23.00-12.50)	0.48
**Pain**	80.18 (67.02-92.39)	81.19 (72.63-91.15)	80.03 (72.75-88.97)	78.36 (70.67-85.66)	75.46 (64.81-86.61)	1.51 (-14.20-16.20)	0.84
**General health**	72.52 (61.53-84.47)	75.67 (65.25-86.51)	75.17 (64.85-84.82)	74.75 (65.17-84.27)	73.86 (61.79-85.61)	3.00 (-10.00-17.00)	0.63
**Change in health**	55.73 (38.60-70.80)	50.66 (40.09-61.67)	52.56 (44.06-62.29)	55.34 (46.69-63.61)	60.53 (45.28-74.89)	-5.74 (-25.80-13.90)	0.56

Data are presented as estimated marginal means (95% confidence intervals) derived from linear mixed-effects models. Each value represents the model-estimated marginal mean for the intervention cohort at the corresponding study timepoint. Treatment effect estimates are shown with corresponding 95% confidence intervals. Treatment effects were estimated using linear mixed-effects models with fixed effects for timepoint and random intercepts for participant.

Supplementary Table 5 Longitudinal changes in RMT and AMTs following Vo-complex thalamotomy

Measure	Timepoint	Estimated marginal mean ± SE	Difference from baseline (95% CI)	*p*-value
**RMT**	**Baseline**	39.27 ± 2.67	N/A	-
	**1 Month**	36.36 ± 2.74	-2.92 (-6.21 to 0.38)	0.08
	**3 Month**	36.87 ± 2.83	-2.40 (-6.01 to 1.20)	0.18
	**12 Month**	37.44 ± 2.71	-1.84 (-5.00 to 1.33)	0.24
**AMT**	**Baseline**	34.18 ± 2.30	N/A	-
	**1 Month**	33.36 ± 2.41	-0.82 (-4.61 to 2.97)	0.66
	**3 Month**	33.24 ± 2.54	-0.94 (-5.08 to 3.20)	0.64
	**12 Month**	31.99 ± 2.35	-2.20 (-5.84 to 1.45)	0.23

Estimated marginal means (± standard error) for pre-PAS resting motor threshold (RMT) and active motor threshold (AMT) at each study timepoint are shown for baseline (n = 11), 1 month (n = 11), 3 months (n = 10) and 12 months (n = 10), derived from separate linear mixed-effects models. Each value represents the model-estimated marginal mean threshold for the intervention cohort at the corresponding study timepoint. Difference from baseline values represent model-estimated changes relative to baseline with corresponding 95% confidence intervals. Pairwise comparisons were performed without adjustment for multiple testing.

Supplementary Table 6 PAS induced reduction of RMT/AMTs in the APB muscle in intervention participants

RMT	Pre	0 min	15 min	30 min	Avg reduction	*p* (Avg vs pre)
**Baseline**	39.27 ± 2.65	36.46 ± 2.65	35.64 ± 2.65	36.12 ± 2.67	-3.20	**< 0.01**
**1 Month**	36.47 ± 2.69	34.80 ± 2.69	35.14 ± 2.69	35.43 ± 2.71	-1.35	0.24
**3 Month**	36.84 ± 2.74	34.27 ± 2.74	35.98 ± 2.74	35.64 ± 2.84	-1.54	0.24
**12 Month**	37.55 ± 2.67	36.25 ± 2.67	34.95 ± 2.67	34.85 ± 2.67	-2.20	**0.04**

AMT	Pre	0 min	15 min	30 min	Avg reduction	p (Avg vs pre)
**Baseline**	34.18 ± 2.08	31.91 ± 2.08	32.18 ± 2.08	33.61 ± 2.10	-1.61	0.08
**1 Month**	33.51 ± 2.12	32.40 ± 2.12	32.51 ± 2.12	32.46 ± 2.15	-1.05	0.30
**3 Month**	33.36 ± 2.18	32.36 ± 2.18	33.22 ± 2.18	33.05 ± 2.27	-0.48	0.68
**12 Month**	32.12 ± 2.10	31.02 ± 2.10	31.52 ± 2.10	32.92 ± 2.10	-0.30	0.75

Values are estimated marginal means ± standard error (% TMS intensity) derived from separate linear mixed-effects models and are shown for baseline (n = 11), 1 month (n = 11), 3 months (n = 10) and 12 months (n = 10). Each value represents the model-estimated marginal mean resting or active motor threshold for the intervention cohort at the corresponding PAS condition and study timepoint. Average reduction represents the model-estimated average difference between pre-PAS and the three post-PAS assessments (0, 15 and 30 minutes) at each study timepoint. *p*-values correspond to the average post-PAS versus pre-PAS contrast and are unadjusted for multiple comparisons.

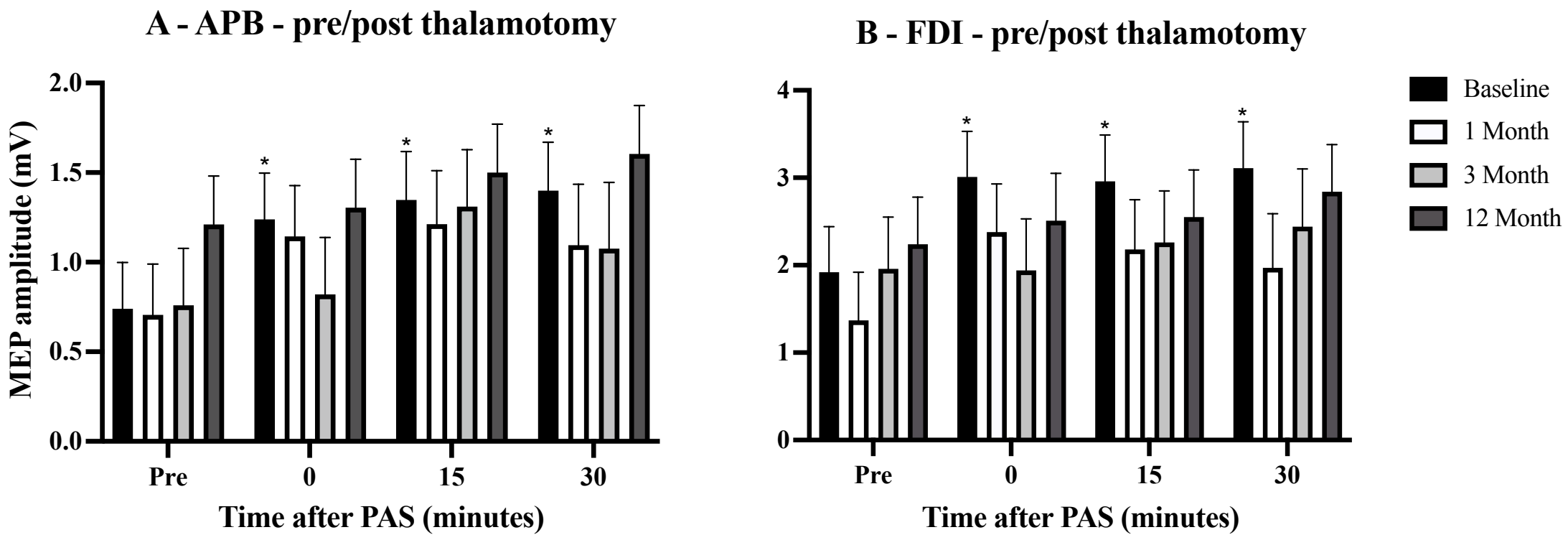

Supplementary Figure 3 PAS induced changes in MEP amplitude before and after Vo-complex thalamotomy. Estimated marginal mean MEP amplitudes (in mV) derived from separate linear mixed-effects models are shown from the (A) APB and (B) FDI muscles at baseline (n = 11) and 1 month (n = 11), 3 months (n = 10) and 12 months (n = 10) post-thalamotomy are shown for pre-PAS and at 0, 15 and 30 minutes after PAS. Each bar represents the model-estimated marginal mean MEP amplitude at the corresponding PAS condition and study timepoint. Error bars represent the standard error of the mean (SEM). MEP amplitudes were analysed using linear mixed-effects models with fixed effects for PAS condition, timepoint, and their interaction, with random intercepts for participant. Significance calculated in comparison to pre-PAS value (* = $p < 0.05$).

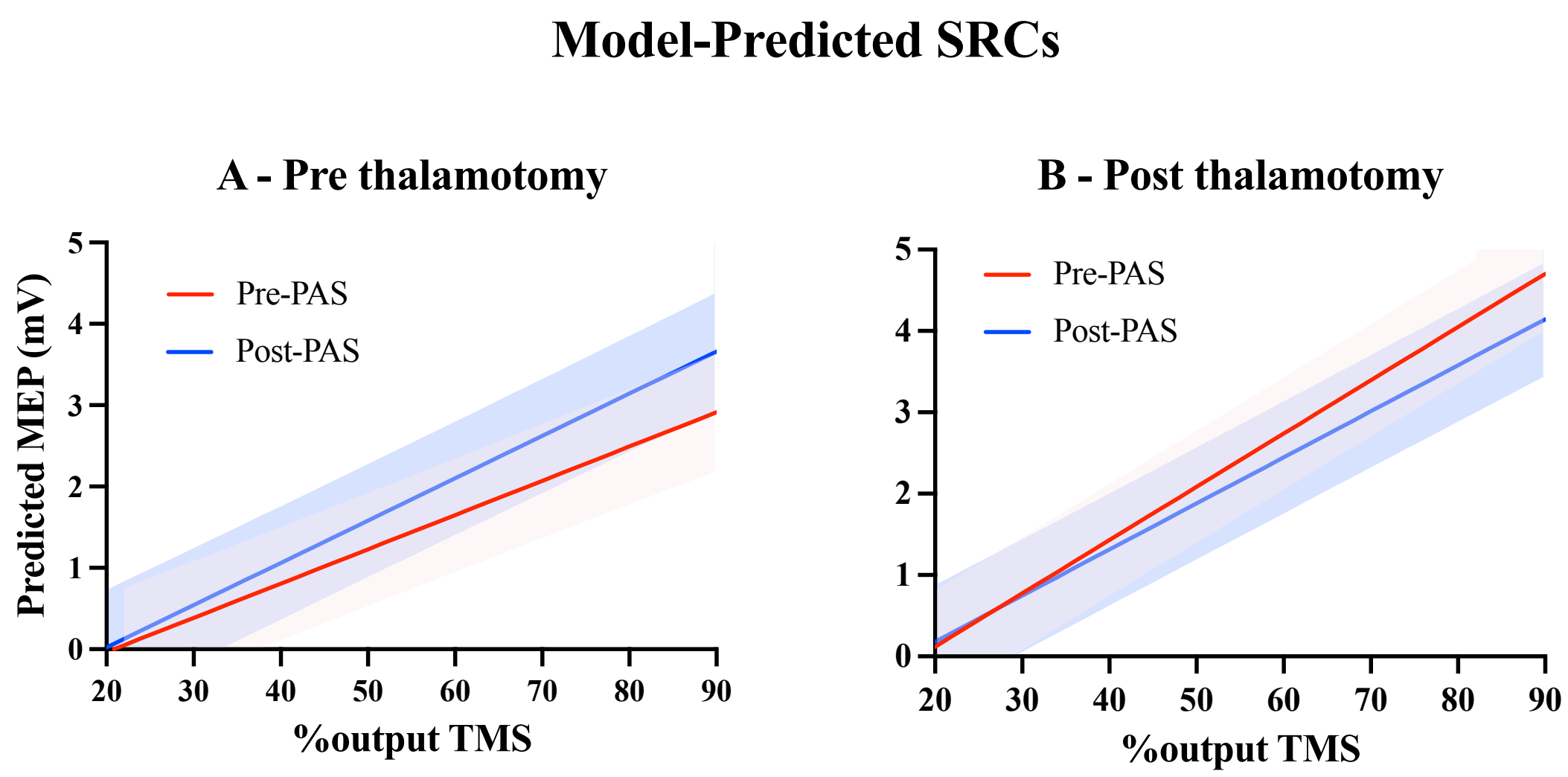

Supplementary Figure 4 Model predicted SRCs illustrate the effect of PAS on corticospinal excitability. (**A**) Prior to thalamotomy (n = 11), post-PAS slopes were steeper than pre-PAS, consistent with facilitation and (**B**) following thalamotomy (n = 10), this effect was attenuated or reversed. The plotted slopes represent the model-estimated marginal mean MEP amplitude at the corresponding stimulation intensity.

Lines represent model-predicted motor evoked potential (MEP) amplitudes derived from linear mixed-effects modelling across the range of stimulation intensities, and shaded areas represent the standard error of the estimated marginal means (SEM). Linear mixed-effects modelling demonstrated a significant three-way interaction between stimulation intensity, PAS condition and timepoint ($F = 7.59$, $p < 0.001$). Estimated marginal trend analysis demonstrated significantly steeper post-PAS slopes prior to thalamotomy ($p = 0.005$).

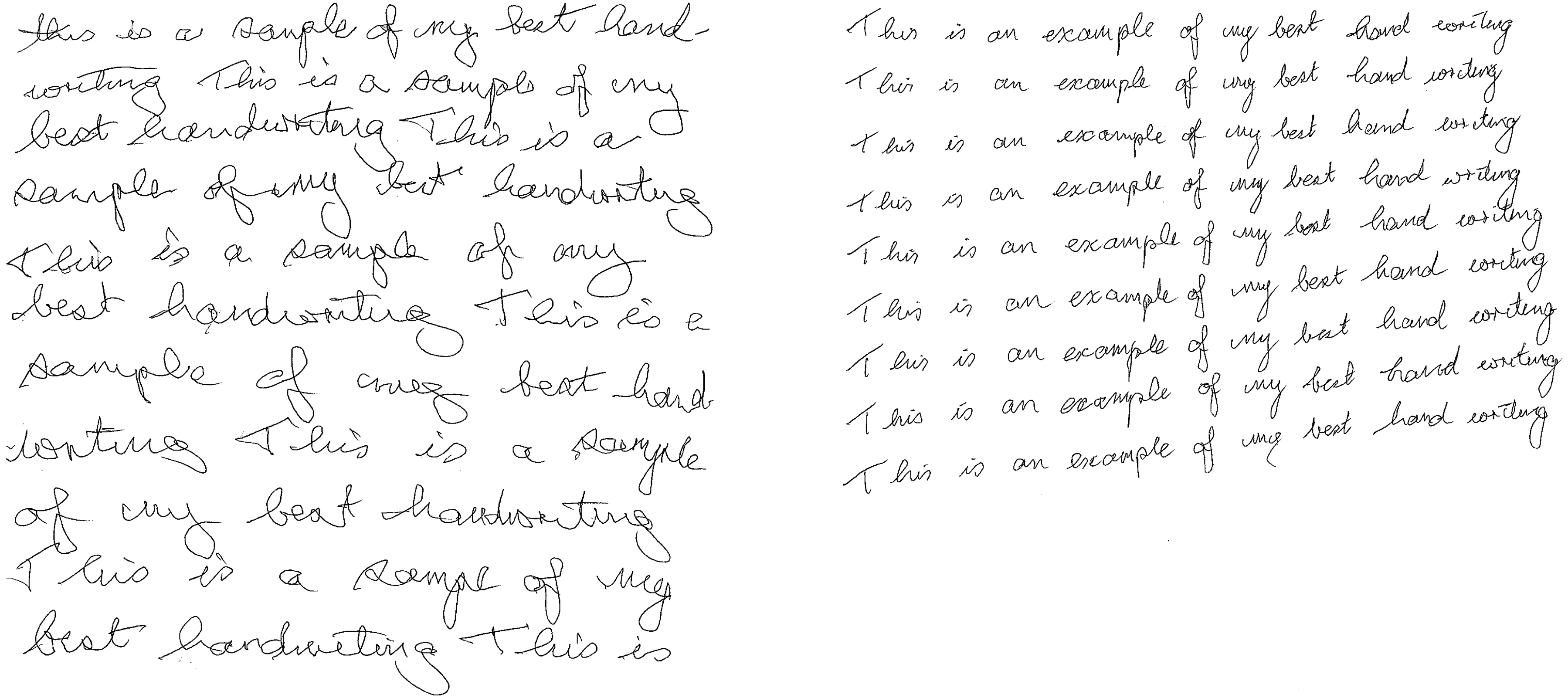

Supplementary Figure 5 Case example of a participants handwriting at baseline (left panel) and 1 month post thalamotomy (right panel). Despite marked improvements in writing quality, the WCRS did not improve (Baseline - 14, 1 month - 14)
